# Severe Case of *Plasmodium falciparum* Malaria in a Pregnant Woman from Nigeria

**DOI:** 10.1155/2019/2630825

**Published:** 2019-10-29

**Authors:** Kruti Yagnik, Bilal Farooqi, Molly W. Mandernach, Anthony P. Cannella, Gautam Kalyatanda

**Affiliations:** ^1^University of Florida, Department of Medicine, Gainesville, FL, USA; ^2^University of Florida, Division of Hematology & Oncology, Gainesville, FL, USA; ^3^University of Florida, Division of Infectious Diseases & Global Medicine, Gainesville, FL, USA

## Abstract

Human malaria has arguably affected more of human history than any other pathogen. Pregnant women have a higher risk of developing severe malaria as well as the risk of severe complications. We present a case of severe malaria in a pregnant patient from sub-Saharan Africa who was treated successfully with artesunate. A 28-year-old Nigerian woman with a 20-week intrauterine pregnancy presented with a five-day history of fever and diffuse joint pains. Evaluation of peripheral thin blood smear demonstrated a parasitemia of 9.8%. The patient was admitted to the intensive care unit, and oral clindamycin/quinine was initiated until intravenous artesunate was obtained. The patient completed four doses of IV artesunate, and after the 4^th^ dose of artesunate, no blood parasites were seen on peripheral smear. The patient was discharged home and, upon clinic follow-up, did not have any further complications associated with either her disease or therapy. A review on the treatment of severe malaria in all trimesters of pregnancy supports the WHO recommendation for intravenous artesunate as the drug of choice. This case illustrates the importance of recognizing malaria in pregnant women from endemic countries and shows that artesunate compounds can be used safely in pregnancy, particularly with high parasitemia.

## 1. Introduction

Human malaria, which had over 200 million documented infected cases in 2016, has arguably affected more of human history than any other pathogen. The genus *Plasmodium* cycles through female Anopheles mosquitos, which then feed on humans and spread the infection, and can in some individuals occur as an asymptomatic infection [1]. While symptomatic cases of *Plasmodium vivax*, *Plasmodium malariae*, and *Plasmodium ovale* have more of an indolent course of infection, *Plasmodium falciparum* has a more fulminant pathogenic response and accounts for the majority of deaths from human malaria, especially in those who are less than five years of age in Africa [2]. Roughly 1,700 cases of malaria are diagnosed in the United States (US) annually, with the overwhelming majority of cases occurring in travelers and immigrants returning from countries where malaria transmission occurs [1]. Severe malaria warrants parenteral antimalarial therapy in a patient and is mandated through criteria from both the clinical presentation (impaired consciousness, distributive shock, pulmonary edema, seizures, and acute respiratory distress syndrome (ARDS)) and laboratory findings (severe anemia, renal failure, lactic acidosis, hypoglycemia and particularly parasitemia >5%) [3]. Pregnant women have a three-fold higher risk of developing severe malaria compared to nonpregnant women, as well as the risk of severe complications such as pulmonary edema, placental infarction, and hypoglycemia [4]. In 2013, thirty-six cases of malaria in pregnant women were reported to the US Centers for Disease Control and Prevention (CDC) of which eight cases were severe [1]. In pregnancy, malaria infection can cause intrauterine growth restriction, low birth weight, and newborn mortality [3]. Severe malaria is a large contributor to maternal morbidity and mortality particularly in developing nations [5]. In the United States, previous parental treatment included a combination of a tetracycline (or clindamycin if the patient is pregnant) and quinidine, yet current recommendations for the use of artesunate-derived compounds are superior at reducing both the parasitemia and the hepatic forms. However, this poses a problem in pregnant women with severe or fulminant malaria, as these compounds may cause teratogenic effects to the fetus based on animal models [5]. Here, we present a case of severe malaria in a pregnant patient from sub-Saharan Africa at our institution that was treated successfully with artesunate without adverse effects.

## 2. Case

**Figure 1:**
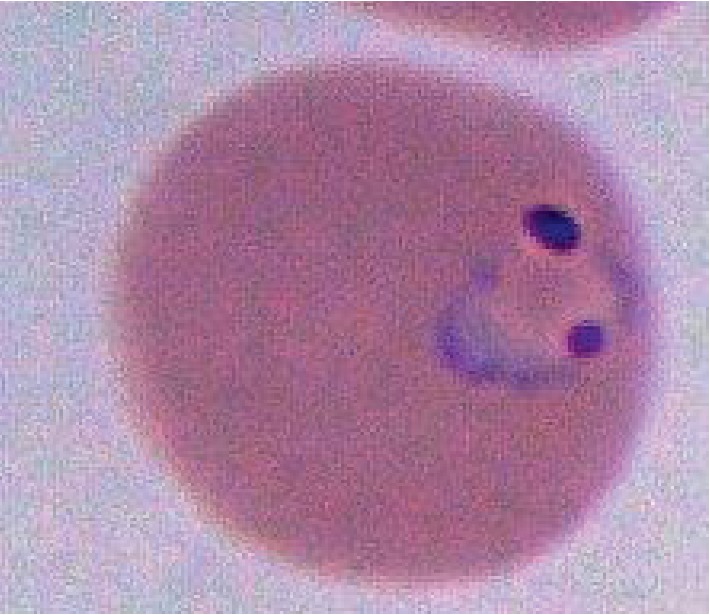
Enlarged picture of a ring-form trophozoite of *Plasmodium falciparum*.

**Figure 2:**
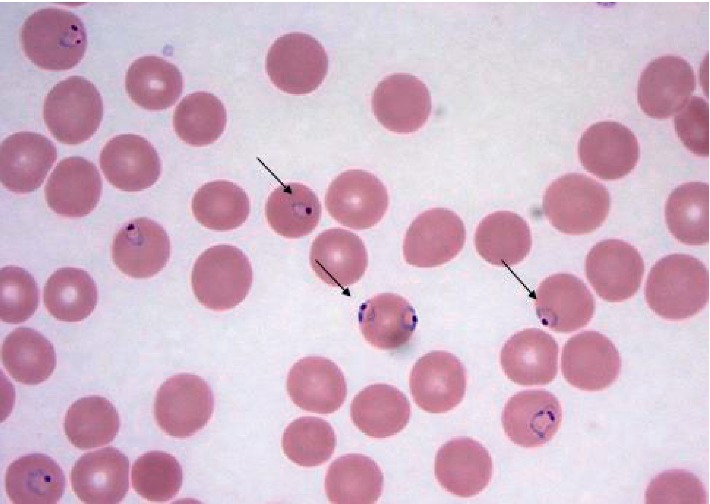
A peripheral thin smear of patient's *Plasmodium falciparum* parasites at admission as indicated by arrows. Slide prepared using Wright stain at 100X magnification.

**Figure 3:**
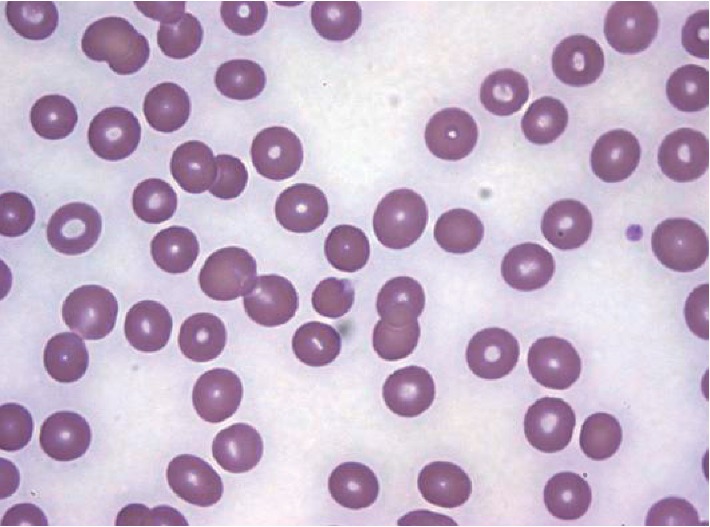
Peripheral thin smear noting near complete resolution of parasites four days after diagnosis. Slide prepared using Wright stain at 100X magnification.

A 28-year-old Nigerian woman with a 20-week intrauterine pregnancy presented to the University of Florida Health Emergency Department (ED) with a five-day history of fever, diffuse joint pain, night sweats, vomiting, and malaise. She had arrived in the US to visit her family about nine days before her admission. She was initially diagnosed with cystitis and *Streptococcus pyogenes* throat infection and discharged with cephalexin for treatment. Her symptoms worsened, and she returned three days later to the ED for evaluation. On physical exam, she was afebrile, tachycardic, tachypneic, and hypotensive. Pertinent initial labs demonstrated hypoproliferative anemia (hemoglobin 8.9 g/dL, was 11 g/dL three days prior, reticulocyte count 0.5%), thrombocytopenia (platelets 39,000/mm^3^, was 101,000/mm^3^ three days prior), D-dimer of 10.04 *μ*g/mL, and LDH 341 U/L. Total bilirubin was 1.2 mg/dl. Complete blood count led to the evaluation of peripheral smear in which numerous inclusion bodies were noted (Figure 1). On further history, she confirmed that she discontinued her malaria prophylaxis, trimethoprim/sulfamethoxazole, due to emesis associated with her pregnancy. A thick blood smear was prepared and showed evidence of malaria parasites, and her BiNax NOW® test was positive for *P. falciparum*. Her thin blood smears (Figure 2) demonstrated a parasitemia calculated at 9.8%, and thus, the patient was admitted to the intensive care unit for close monitoring. Oral clindamycin and quinine were initiated until the CDC delivered intravenous artesunate, which was then continued. Her hospitalization was complicated by significant hypoglycemia requiring 5% dextrose infusion, and severe dyspnea was thought to be due to pulmonary edema requiring intravenous furosemide diuresis. CTA was negative for pulmonary embolism. The first dose of artesunate was given at 18:45 hours on day 2, and by 12:00 on day three, the parasite load was 0.09%. Patient completed four doses of IV artesunate and seven days of oral clindamycin. After the 4^th^ dose of artesunate, no blood parasites were seen on peripheral smear (Figure 3). The patient was discharged home after a nine-day hospitalization with improvement in her anemia and thrombocytopenia. Upon clinic follow-up a few weeks later, the patient did not have any further complications associated with either her disease or therapy, with normal labs and an uncomplicated remainder of her pregnancy.

## 3. Discussion

It should be noted that pregnant women in Nigeria (and other parts of sub-Saharan Africa) are usually given intermittent preventive treatment for malaria. Our patient was unable to tolerate prophylactic trimethoprim/sulfamethoxazole secondary to chronic emesis; this is of great importance as pregnant women are noted to have an increase in gametocytemia (mainly in the placenta) and thus have an increased risk of transmitting malaria to others who live in the same vicinity due to this increased gametocyte reservoir [6]. The WHO recommends treatment of *P. falciparum* malaria in both the second and third trimesters the same as for nonpregnant adults. A review on the treatment of severe malaria in all trimesters of pregnancy supports the WHO recommendation for intravenous artesunate as the drug of choice [7].

Malaria due to *P. falciparum* causes intrauterine growth retardation, premature delivery, and low birth weight and thus increases newborn mortality; it also increases maternal morbidity and mortality. Peripheral smears may be negative due to sequestration within the placenta. Selective accumulation of infected erythrocytes in the placenta involves their interaction with syncytiotrophoblastic chondratin sulfate A. In regard to complete blood counts, severe malaria is known to cause both anemia and thrombocytopenia [8]. Malaria can induce anemia through T cell- dependent parasite clearance [9]. Thrombocytopenia has been noted to be more common in pregnant women with malaria as opposed to nonpregnant women [9]. Thrombocytopenia in malaria is attributed to decreased lifespan, splenic sequestration, and platelet activation [10, 11].

The CDC guidelines for treatment of severe malaria include parenteral quinidine gluconate plus either doxycycline, tetracycline, or clindamycin. Artesunate-based parenteral therapy followed by an oral course (atovaquone-proguanil, doxycycline, or mefloquine) can also be used [1]. In the US, artesunate is only obtained from the CDC even though this medication is the preferred therapy by WHO and countries where malaria is endemic [3]. Our patient received clindamycin and oral quinine due to unavailability of IV quinidine until artesunate was obtained from the CDC. In two studies comparing intravenous quinine with intravenous artesunate, intravenous artesunate demonstrated efficacy and safety in pregnant women [5]. There was no increased risk of miscarriage, stillbirth, or congenital anomalies associated with first-trimester exposure to artesunate [5].

This case illustrates the importance of recognizing malaria in pregnant women from endemic countries and that artesunate compounds can be used safely in pregnancy particularly in individuals with high parasitemia.
